# An Illustrative Approach to Removing Conjunctival Bitten Ant Foreign Body

**DOI:** 10.7759/cureus.68038

**Published:** 2024-08-28

**Authors:** Shubhangi SN Prasad, Padmaja Nama, R Balamurugan, Mohan Kumar A

**Affiliations:** 1 Ophthalmology, All India Institute of Medical Sciences Mangalagiri, Mangalagiri, IND; 2 Microbiology, All India Institute of Medical Sciences Mangalagiri, Mangalagiri, IND; 3 Medical Microbiology, All India Institute of Medical Sciences Mangalagiri, Mangalagiri, IND

**Keywords:** safe removal of ant from eye, conjunctival bitten ant foreign body, removal of red ant in the eye, ocular insect removal method, ocular animate foreign body, conjunctival foreign body, ocular foreign body, forceps removal, ocular emergencies, ant foreign body

## Abstract

An animate ocular foreign body poses a challenging situation for the ophthalmologist for its complete removal. Incomplete removal may lead to infection and inflammation. A woman in her 50s presented with a history of an ocular foreign body in her left eye while working in the agricultural land. On examination, there was conjunctival congestion and a copious amount of thick secretion in the inferonasal region of the conjunctiva. There was a dark black dot in the red body embedded in the secretion, which aroused my suspicion of an animate object (the red ant). In an animate ocular foreign body, complete removal is warranted to prevent infection and inflammation. Hence, the thick secretion that hid the animate object's body was gently removed using cotton tip applicators, and a part of the dead red ant bitten to the conjunctiva was visible. Initially, we tried to remove it using a cotton tip applicator but failed. In order to remove it completely, we tried to remove it using plain forceps, which were successfully removed by grabbing its head under a slit-lamp examination. The ant was sent to the parasitology department, which ensured the complete removal of the ant in the presence of its mandible. The patient was completely relieved of the symptoms and signs at the one-week follow-up. In this case report, we illustrate our stepwise approach to the management of a conjunctival bitten dead ant foreign body for its complete removal with a photo and video demonstration.

## Introduction

An ocular foreign body is an emergency condition that needs prompt removal to mitigate the patient's suffering and its complications. The ocular foreign body could be an inanimate object (dust, sand, metal, etc.) or an animate or organic object like ants, tarantula hairs, caterpillar setae, beetles, etc. [1,2]. Removal of animate objects like insects needs to be very careful because incomplete removal leads to an allergic reaction to their left-over remnant [1,2]. We report an unusual case of a dead ant that was found to have bitten the conjunctiva, and we describe our stepwise approaches to removing the bitten dead ant completely without leaving any of its remnants in the eye.

## Case presentation

**Figure 1:**
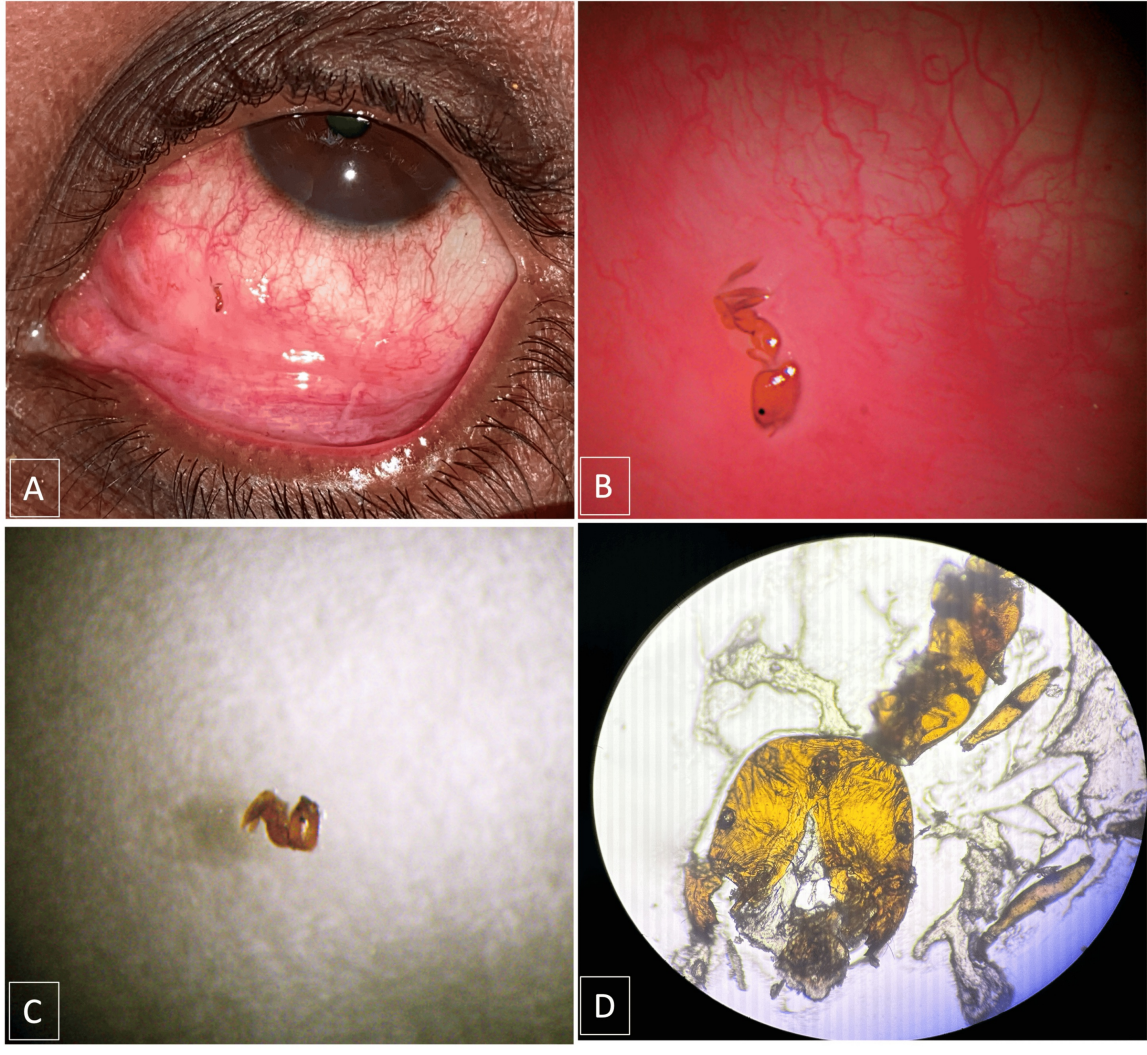
(A-D): 1A shows the naked eye appearance of an ant foreign body in the eye after the adequate removal of the thick secretions. 1B shows a magnified image of a dead red ant bitten to the conjunctiva under slit lamp examination. 1C shows the head of the ant was not crushed after removal from the conjunctiva using plain forceps, but the intactness of the mandible was not visible under slit lamp magnification. 1D shows that the light microscopy of the specimen shows the presence of both the mandibles of the ant, ensuring its complete removal from the conjunctiva.

A female in her 50s presented with a one-day history of redness and a foreign body sensation following a foreign body that fell in her left eye while working on agricultural land. Her daughter found a foreign body in the eye and attempted removal using splashing water and also by using cloth, but it failed. The symptoms were worsening, and hence she presented to the hospital the next day. On examination, both her eyes' visual acuity was 20/20 (unaided) with normal intraocular pressure. She had intense watering with conjunctival redness and eyelid swelling in her left eye. On slit lamp examination, her left eye conjunctiva shows diffuse congestion along with copious amounts of thick secretion covering the inferotemporal quadrant of the conjunctiva. There was a black dot in a red body embedded in the secretions, which gave me a clue about the head of an ant. In view of the suspicion of an ant foreign body, the secretion material was gently removed using the sterile cotton buds without disturbing the foreign body. After adequate removal of the secretion material, a red ant was found to have bitten the conjunctiva with localized chemosis (Figure 1A-1B). The ant was found to be dead, with half of its body parts missing.

Under topical anesthesia (proparacaine 0.5%), we tried to remove it gently using a sterile cotton tip applicator but failed. Our main aim is to remove the bitten ant completely without leaving any of its parts, especially its mandible, to avoid an inflammatory reaction. We used plain forceps to remove it by gently grabbing its head and gently pulling it out of the conjunctiva (Video 1). There were no remnants visible under 16X slit lamp magnification. Injection tetanoid toxoid was given intramuscularly, and prophylactic topical antibiotics (moxifloxacin 0.5%) were given six times a day for one week.

**Video 1:** Removal of a conjunctival bitten ant foreign body

The ant was placed on the glass slide and examined (Figure 1C). Its head was not crushed while being removed using metal plain forceps, but the intactness of the mandible was not visible (Figure 1C). Hence, the extracted ant was sent to the parasitology department to ensure the presence of its mandible. The sample was sent on glass slides mounted on another glass slide. Under 10X magnification, the ant head was seen as crushed while transported in the glass slides, but the presence of both mandibles ensured the complete removal of the ant from the conjunctiva (Figure 1D).

The patient was followed up the next day with no symptoms and a complete disappearance of the secretions. Conjunctival congestion and chemosis decreased. Hence, the antibiotics were continued for a complete week and then advised to stop.

## Discussion

Ocular foreign bodies could be due to the animate objects, which need a separate strategy to be removed completely, for example, injection of suxamethonium into the leech or direct application of cooking salt onto it for leech removal [3]; using plan forceps to remove oestrus ovis larvae from the conjunctiva [4]; and using a bent 30-gauge needle used to remove the ant bitten the conjunctiva [2]. Incomplete removal of the animate objects may lead to anaphylactic reactions. Lim [2] attempted many methods, like cotton tip applicators and plain non-toothed forceps, to remove the conjunctival bitten ant, but they were unsuccessful. However, they removed it by using a 30-gauge bent needle atraumatically. We have also attempted to remove the ant by using a cotton tip applicator first, but it was unsuccessful. Hence, we used plain forceps only to remove this bitten ant, which was successfully removed completely. Care should be taken not to crush the head of the ant while removing it.

## Conclusions

An ocular animate foreign body may induce intense inflammation with thick secretions, which may hide the inciting animate objects; hence, gentle removal of the slough material using a cotton tip applicator or irrigation is recommended to make the inciting primary animate objects undisturbed. Complete removal of the animate objects is of utmost importance to avoid infections and allergic reactions. After removal, the animate foreign body should be sent for light microscopy for proper identification as well as to ensure its complete removal. Incomplete removal should be closely watched or may require further surgical intervention if the inflammation persists.
